# Navigating Prevention: A Systematic Review of Strategies for Alzheimer’s Disease in High-Risk and Affected Individuals

**DOI:** 10.7759/cureus.65972

**Published:** 2024-08-01

**Authors:** Carlos Luis Guanín Cabrera, Arturo P Jaramillo, Maria P Vallejo, Maria G Cueva, Xavier Grandes, Mario Navarro Grijalva, Over J Hidalgo Guerrero

**Affiliations:** 1 General Practice, Universidad Católica de Santiago de Guayaquil, Guayaquil, ECU; 2 General Practice, Universidad Estatal de Guayaquil, Machala, ECU; 3 Urology, Universidad Catolica de Santiago de Guayaquil, Guayaquil, ECU; 4 Research, Universidad Catolica de Santiago de Guayaquil, Guayaquil, ECU; 5 Internal Medicine, Universidad Catolica de Santiago de Guayaquil, Guayaquil, ECU

**Keywords:** neuroprotector, mind diet, dementia, preventive medicine, alzheimer’s dementia

## Abstract

Alzheimer's disease (AD) remains a widespread cause of dementia globally, and its prevalence is increasing due to the aging population. Two key pathologies typically identify this neurodegenerative disease process: the accumulation of amyloid plaques and the formation of neurofibrillary tangles containing hyperphosphorylated tau. Diagnosis relies on the patient's clinical presentation meeting specific criteria, along with the use of fluid and imaging biomarkers. The current treatment focuses on addressing symptoms, with ongoing trials aiming to decrease the production and overall impact of brain pathology. Here, we explore various methods to minimize the risks of AD in patients and individuals at high risk of developing it. To address this, we carefully selected 10 articles that discuss various prevention methods used today to promote brain health, including diets that are believed to have neuroprotective properties. The study findings emphasize the importance of further strengthening the evidence and conducting larger randomized controlled trials to gain a better understanding of the potential benefits for individuals at high risk of developing AD, as well as those already diagnosed with it.

## Introduction and background

In the coming decades, there is expected to be a significant increase in the number of individuals aged 60 years and older, reaching a staggering 1.25 billion. This demographic shift will also bring about a rise in the number of people affected by dementia, estimated to be around 115.4 million [1]. Alzheimer's disease (AD) is a common form of dementia, affecting the majority of cases. It is characterized by the presence of intraneuronal fibrillary tangles and extracellular amyloid plaque (Aβ) deposits. These changes are accompanied by reactive microgliosis, neuronal loss, and cortical dysfunction [2]. These Aβ deposits can lead to severe cognitive impairments, such as memory and intellectual disabilities, which can greatly affect daily activities and diminish the overall quality of life [2]. While current pharmacological treatments may provide some relief from symptoms, they do not address the underlying disease. This emphasizes the importance of exploring new non-pharmacological interventions [3].

People with mild cognitive impairment (MCI) experience cognitive changes that go beyond what is typical for their age and education level, but these changes do not greatly disrupt their daily activities. Nevertheless, MCI has been linked to a higher likelihood of developing dementia, with a prevalence ranging from 9.6% to 21.6% worldwide [4]. Pharmacological treatments for MCI have demonstrated limited effectiveness, highlighting the need for innovative therapeutic strategies [5]. It is highly advised to refrain from copying others' work in order to maintain academic integrity. Plagiarism can have serious consequences and should be avoided at all costs. Instead, it is recommended to engage in cognitive stimulation as a non-pharmacological intervention for cognitive symptoms in MCI and mild-to-moderate dementia. However, it is important to note that the evidence supporting cognitive training is still in its early stages [6].

Engaging in physical activities (PAs) has shown promise as a non-pharmacological approach to treating dementia. Research has indicated that PAs may help slow down cognitive decline in older adults who are in good health. However, the impact of PA on cognitive decline is not always consistent, mainly because of limitations in the methods used, such as different types of exercise interventions and small participant groups. Research has shown that engaging in aerobic and resistance PAs can have a positive impact on cognitive functions such as attention, memory, and executive functions in individuals with MCI. However, no cognitive benefits were observed in individuals with AD [7]. Past research has indicated that physical activity treatment (PT) has the potential to improve overall cognitive function and memory in individuals with MCI, as well as slow down cognitive decline in those who are at risk of or have been diagnosed with AD [7]. However, it is important to conduct additional research using more specific neuropsychological measurements due to the differences between studies and outcomes.

The purpose of this study is to examine the impact of cognitive treatment (CT) and PAs on older adults with AD and MCI. There is a hypothesis that both CT and PA could potentially slow down the cognitive decline in individuals with AD and MCI. These interventions have similar primary outcomes but may differ in their secondary outcomes. It is anticipated that CT will have a positive impact on memory, while PT is likely to improve physical function and attention.

Yoga has demonstrated significant advantages for improving cardiovascular health and reducing stress levels, making it a valuable approach to target certain factors that can be modified to reduce the risk of AD [8]. Recent studies have found that yoga can be a beneficial and safe activity for improving cognitive functions in older adults, including those with MCI and early-stage dementia [9]. Research has shown that practicing yoga can have positive effects on the mental and physical well-being of older adults with dementia in long-term care facilities. It has been found to improve various aspects of health, such as blood pressure, breathing rate, cardiorespiratory fitness, body flexibility, balance, joint movement, muscle strength, and endurance [10]. General population studies also suggest that people practice yoga for various reasons, such as enhancing overall well-being, preventing diseases, improving fitness and energy levels, promoting physical and mental health, boosting immune function, and relieving conditions like back pain, arthritis, anxiety, and depression [11].

Yoga often incorporates various elements such as postures, breath control, postural alignment, and movement. In addition, incorporating short moments of mindfulness can be advantageous. For instance, in subjective cognitive decline, a yogic meditation known as Kirtan Kriya (KK) showed significant improvements in perceived stress, psychological well-being, quality of life, and mood when compared to a control group that listened to music [8,11]. Yoga is widely recognized for its positive impact on stress reduction, memory enhancement, brain health, and overall well-being. It is particularly beneficial for high-risk groups and the general population alike.

Studies have indicated that individuals who regularly practice yoga tend to have higher overall gray matter volume (GMV) in certain areas of the brain, such as the left insula, frontal operculum, and orbitofrontal cortices. This correlation becomes stronger with longer experience in yoga [11]. It was discovered in another study that regular yoga practice can lead to increases in GMV in various regions of the brain, including the primary somatosensory and superior parietal cortices, precuneus, posterior cingulate junction, hippocampus, and primary visual cortex. These findings indicate that practicing yoga could potentially provide benefits for brain health. In addition, individuals who have been practicing yoga for a long time showed a larger volume in the left hippocampus when compared to individuals who are new to yoga and are of the same age and sex [11]. Yogic meditation practitioners experienced an increase in GMV in various regions of the brain, including the frontal, limbic, temporal, occipital, and cerebellar regions. This increase is linked to improved cognitive performance [11]. In addition, it was found that experienced yoga meditators showed higher global GMV compared to those who did not meditate. This was especially evident in specific areas of the brain, such as the right ventromedial orbitofrontal and ventrolateral prefrontal cortices, inferior temporal and parietal cortices, and bilateral insula cortex [11].

A four-week Sahaja yoga meditation training resulted in increased activity in the right inferior frontal gyrus and improvements in well-being, fatigue, and satisfaction when compared to a waitlist control group [11]. In a small, uncontrolled six-month yoga trial involving older, healthy volunteers, there were reported increases in hippocampal volume. However, no changes were observed in a control region in the occipital cortex [11]. The main objective of this systematic review is to thoroughly analyze the most recent studies on different forms of preventive medicine utilized by patients diagnosed with AD and those at high risk of developing it.

## Review

Methods

Review Records and Search for Studies

This systematic review adhered to the guidelines of Preferred Reporting Items for Systematic Reviews and Meta-Analyses (PRISMA) [12]. The article selection process involved independent researchers conducting comprehensive searches in PubMed, PubMed Central, and other databases. Details of the search methodology employed can be found in Table 1.

**Table 1:** Search strategy for databases

Search Strategy	Databases Used	Number of Papers Identified
Alzheimer’s Disease AND Preventive Medicine AND High Risk for Alzheimer’s Disease	PubMed	133
( "Alzheimer Disease/etiology"[Majr:NoExp] OR "Alzheimer Disease/prevention and control"[Majr:NoExp] OR "Alzheimer Disease/rehabilitation"[Majr:NoExp] OR "Alzheimer Disease/therapy"[Majr:NoExp] )	PubMed Central (PMC)	2102
"Alzheimer’s Disease [tw]" AND "Preventive Medicine [tiab]" AND "High Risk for Alzheimer’s Disease [all]"	Cochrane Library	334

Inclusion and Exclusion Criteria

Two independent authors utilized the Covidence software to screen the search results obtained from two databases following pre-established inclusion and exclusion criteria, shown in Table 2.

**Table 2:** Inclusion and exclusion criteria

Inclusion	Exclusion
Free, full text about Alzheimer’s disease and those with a high risk of developing the disease	Articles that include> 95-year-old patients
Articles from the past 10 years	Articles from 2013 and below
English-language articles	Non-English studies
Prospective or retrospective studies	Case reports
Human trials	Animal trials

Data Extraction

During a thorough examination of the relevant research, several noteworthy discoveries emerged. These include the design of each trial, looking forward to selecting the most commonly practiced preventive methods for AD and those with a high risk of developing the disease.

Risk of Bias Assessment

For the purpose of determining whether or not the studies that were chosen for our investigation had any possible biases, we used the Cochrane risk of bias tool, which was developed especially for randomized controlled trials (RCTs). In the process of assessing the quality of case-series research, this instrument has garnered widespread recognition for its efficiency [13]. Reviewers impartially evaluated the potential for bias in each research and resolved any discrepancies in their assessments through in-depth conversations.

Results

**Figure 1:**
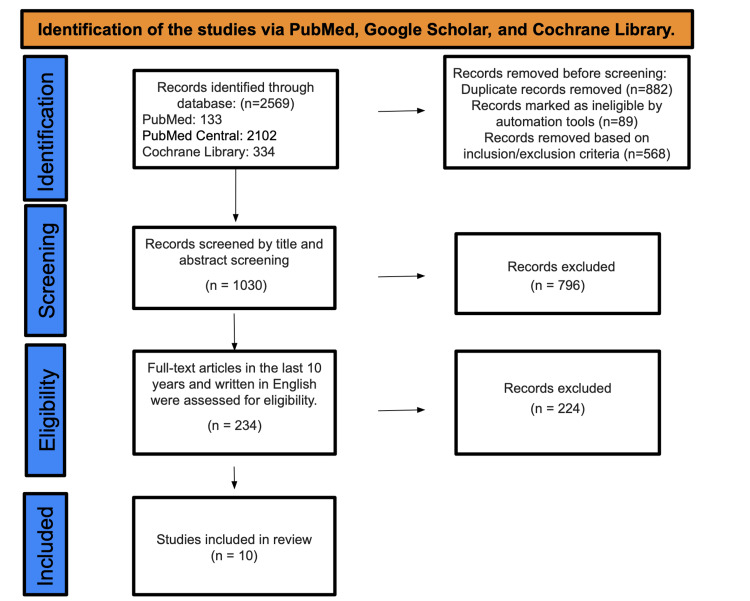
PRISMA diagram PRISMA: Preferred Reporting Items for Systematic Reviews and Meta-Analyses

A total of 2569 studies were found after searching in PubMed, PMC, and Cochrane Library databases. A total of 568 were marked as ineligible based on inclusion and exclusion criteria, and 882 duplicate studies were removed. There were a total of 1030 studies that underwent title and abstract screening, with 796 papers being discarded since they were not related to the purpose of our study. The remaining 234 papers were chosen by their content in English and full-free text evaluation in the previous 10 years, resulting in the elimination of 224 studies; only 10 studies were enlisted for the final collection of data. Figure 1 shows identification of studies via databases and registers.

A list of 14 potential articles on preventive strategies for AD in high-risk and affected individuals was compiled from a literature search, forming the basis of the study's questionnaire. The Delphi technique was used to achieve expert consensus on including several of these identified topics, emphasizing the importance of preventive strategies for treating and preventing Alzheimer's in these populations. The stability of responses across rounds was verified using the chi-square test, with the consensus percentages from Round 1 serving as the observed range and those from Round 2 as the expected range. The resulting P-value of 0.0006 indicated highly stable response data. Table 3 lists the needs and topics identified in the literature search that were presented to the expert panel, with the final agreement percentages in the third column.

**Table 3:** Questionnaire topics MIND: Mediterranean-DASH Intervention for Neurodegenerative Delay

Discipline	Topics	Consensus%
Neurology	Comparison between physical and cognitive treatment in patients with MCI and Alzheimer's disease [7]	88.5
	Yoga prevents gray matter atrophy in women at risk for Alzheimer's disease: a randomized controlled trial [11]	95.2
	Memory support training and lifestyle modifications to promote healthy aging in persons at risk for Alzheimer's disease: a digital application supported intervention (Brain Boosters) [14]	78.6
	Impact of yoga versus memory enhancement training on hippocampal connectivity in older women at risk for Alzheimer's disease [15]	71.5
	Properties of the cognitive function battery for the MIND Diet intervention to prevent Alzheimer's disease [16]	93.2
Geriatrics	36-month LipiDiDiet multinutrient clinical trial in prodromal Alzheimer's disease [17]	78.2
	Randomized crossover trial of a modified ketogenic diet in Alzheimer's disease [18]	83.2
Preventive Medicine	Effects of aerobic exercise on mild cognitive impairment: a controlled trial	65.5
	Comparative efficacy of active group music intervention versus group music listening in Alzheimer's disease [19]	83.7
Physical Medicine	The fitness for the Ageing Brain Study II (FABS II): protocol for a randomized controlled clinical trial evaluating the effect of physical activity on cognitive function in patients with Alzheimer's disease	69.2
	Accuracy of clinical diagnosis of Alzheimer's disease	60.4
Family Medicine	Effects of aerobic exercise training on systemic biomarkers and cognition in late middle-aged adults at risk for Alzheimer's disease [20]	96.4
	A benefit-finding intervention for family caregivers of persons with Alzheimer disease: study protocol of a randomized controlled trial	52.9
	Dietary supplementation with curcumin reduce circulating levels of glycogen synthase kinase-3β and islet amyloid polypeptide in adults with high risk of type 2 diabetes and Alzheimer's disease [21]	95.5
* Strikethrough: topics that remained excluded after the last Delphi round.		

Out of the 14 topics, 11 were agreed upon in the first round, and an additional 10 of the remaining 11 topics were included in the second round. Analysis of the panel's explanations revealed three main reasons for topic inclusion: (i) The most common reason for including a topic was its publication within the last 10 years; (ii) topics that closely aligned with the study's goals for developing the SRL had high inclusion percentages; (iii) items addressing previously neglected needs were also frequently included due to the lack of existing recognition or efforts to address these needs. Table 4 shows an in-depth description of the articles we decided to use.

**Table 4:** Table of data extraction RCT: Randomized clinical trial; SCD: subjective cognitive decline; KY: Kundalini yoga; MET: memory enhancement training; MRI: magnetic resonance imaging; MIND: Mediterranean-DASH Intervention for Neurodegenerative Delay; SD: standard deviation; KY + KK: Kundalini Yoga and Kirtan Kriya; AD: Alzheimer's disease; WRAP: Wisconsin Registry for Alzheimer’s Prevention; WADRC IMPACT: Investigating Memory in People At Risk, Causes and Treatments cohort of the Wisconsin Alzheimer’s Disease Research Center; CTSB: Myokine Cathepsin B;  GSK-3β: Glycogen Synthase Kinase-3 β; IAPP: islet amyloid polypeptide; CT and PA: CT cognitive and physical activity treatments; MMSE: Mental State Examination: MMSE; CTRL: control group; MCI: mild cognitive impairment

Author	Year of publication	Study design	Primary research	Outcome evaluation
Fonte et al. [7]	2019	RCT	For a six-month period, we randomly assigned 87 patients into three groups: CT with 30 participants, PT with 27 participants, and a CTRL with 30 participants. The assessment of global cognitive function among these individuals was conducted using the MMSE.	This research validates the beneficial impacts of CT and PT on reducing cognitive deterioration in patients with MCI and AD. It is also pioneering in showing that both types of training are equally effective in preserving cognitive abilities.
Krause-Sorio et al. [11]	2022	RCT	Eleven women, averaging 61 years old with a SD of 6.58, who had cardiovascular risk factors and subjective cognitive decline, participated in a twelve-week KY + KK program. Concurrently, another group of eleven women, with an average age of 64 years and a SD of 6.41, engaged in MET.	Yoga instruction could potentially provide neuroprotective benefits that surpass those of MET in safeguarding against neurodegenerative alterations and cognitive function deterioration, even for short periods of time. Upcoming studies will investigate alterations in functional connectivity among participants in both groups.
Tomaszewski Farias et al. [14]	2023	RCT	The study involves 225 adults, all 65 years of age or older, who live independently within the community. Notably, 25% of these participants belong to underrepresented groups. Each participant reported experiencing SCD and initially exhibited low engagement in healthy lifestyle behaviors.	If goals 1, 2, and 3 are met, it will be proven that combining training compensation with changes to a person's lifestyle can improve their cognitive abilities and daily functional abilities compared to a group that only receives educational interventions.
Kilpatrick et al. [15]	2023	RCT	The study participants were women who reported subjective memory decline and had cardiovascular risk factors. These individuals were part of a larger RCT that lasted 12 weeks and compared the effects of KY versus MET. All participants underwent MRI scans both before and after the intervention period.	MET may be better at improving hippocampal sensory integration, while KY training may be better at improving stress-related hippocampal connectivity. This could lead to better memory reliability in women who are losing their memories and have heart disease risk factors.
Krueger et al. [16]	2022	RCT	The MIND cognitive function battery was initially conducted on 604 participants, each with an average age of 70, who consented to take part in the dietary intervention study. This battery was specifically crafted to track changes in cognitive function over time.	The findings suggest that the MIND cognitive battery effectively and comprehensively assesses four distinct areas of cognitive function, making it a suitable tool for evaluating dietary interventions in elderly populations.
Soininen et al. [17]	2021	RCT	This RCT enrolled 311 individuals who met the International Working Group-1 criteria for prodromal AD. Participants were randomly allocated to receive either an active product—a 125-mL daily drink—or a control drink that was isocaloric and matched in taste but contained no active ingredients.	This comprehensive multinutrient intervention demonstrated a deceleration in the progression of symptoms associated with cognitive decline, functional abilities, brain shrinkage, and overall disease progression. Prolonged usage of the intervention enhances its benefits, according to the findings.
Phillips et al. [18]	2021	RCT	The study randomized 26 patients, of whom 21 (81%) successfully completed the ketogenic diet regimen. Only one participant discontinued the diet due to issues related to the diet itself. Throughout the study, participants on the ketogenic diet maintained sustained physiological ketosis.	Individuals who followed the ketogenic diet experienced improvements in daily functioning and quality of life, both of which are critical aspects for those with dementia.
Gómez-Gallego et al. [19]	2021	RCT	The study enrolled 90 AD patients from six different nursing homes.	Active music intervention has been shown to enhance AD symptom management and could be effectively incorporated as an adjunct to standard treatment protocols.
Gaitán et al. [20]	2021	RCT	25 middle-aged adults from the WRAP, or the WADRC Impact, and 23 completed the trial.	Following a 26-week structured aerobic exercise program, adults at risk for AD exhibited elevated levels of plasma CTSB. Furthermore, there was a positive correlation between changes in CTSB levels and improvements in cognitive function.
Thota et al. [21]	2020	RCT	Blood plasma samples were collected from participants enrolled in a RCT that involved administering 180 mg/day of curcumin over a period of 12 weeks. These samples were then analyzed to measure levels of circulating GSK-3β and IAPP.	Consuming dietary supplements containing curcumin has been shown to decrease the circulating levels of IAPP and GSK-3β. This finding introduces a new potential pathway by which curcumin might help to lower markers associated with insulin resistance, thereby possibly reducing the risk of T2D and AD.

Table 5 shows the Cochrane risk of bias tool for RCTs.

**Table 5:** Cochrane risk of bias tool

Studies	Random sequence generation (selection bias)	Allocation concealment (selection bias)	Blinding of participants	Blinding of personnel/care providers (performance bias)	Blinding of outcome assessor (detection bias)	Incomplete outcome data (attrition bias)	Selective reporting (reporting bias)	Other biases	Overall
Fonte et al. [7]	+	+	+	+	+	+	+	-	7/8
Krause-Sorio et al. [11]	+	+	+	+	?	+	+	-	6/8
Tomaszewski Farias et al. [14]	+	+	+	+	?	+	+	-	6/8
Kilpatrick et al. [15]	+	+	+	+	+	+	+	-	7/8
Krueger et al. [16]	+	+	+	+	+	+	+	-	7/8
Soininen et al. [17]	+	+	+	+	?	+	+	-	6/8
Phillips et al. [18]	+	+	+	+	?	+	+	-	6/8
Gómez-Gallego et al. [19]	+	+	+	+	+	+	+	-	7/8
Gaitán et al. [20]	+	+	+	+	-	+	+	-	7/8
Thota et al. [21]	+	+	+	+	+	+	+	-	7/8

Discussion

In our systematic review of literature, we have curated a diverse collection of studies exploring the multifaceted benefits of various interventions aimed at delaying or preventing AD. This review includes an analysis of 10 distinct articles, each examining different aspects such as physical exercise, diets like the Mediterranean-DASH Intervention for Neurodegenerative Delay (MIND) diet, the ketogenic diet, and the use of supplements like turmeric.

Additionally, the review covers disciplines like yoga and even music therapy, providing a comprehensive preventive perspective for individuals with AD or those at high risk of developing the condition. The RCT by Tomaszewski et al. highlights the potential benefits of lifestyle interventions in enhancing cognitive function and daily living activities compared to an education-only control group [14]. The study not only focuses on primary outcomes like cognition and daily functionality but also examines secondary outcomes including well-being, physical function, and lifestyle activities. The insights gained from this study aim to refine future programs by identifying the most beneficial aspects of lifestyle interventions for individuals experiencing subjective cognitive decline [14]. In a similar vein, research by Kilpatrick et al. indicates that Kundalini Yoga combined with Kirtan Kriya (KY+KK) may enhance connectivity in the hippocampal regions associated with stress and episodic memory decline, particularly in women with subjective memory issues and cardiovascular risk factors [15]. This study underscores yoga as a beneficial practice for brain health, particularly for older women at elevated risk of AD, by reducing stress and potentially improving memory functions [15].

The study by Krueger et al. sheds light on the interplay between cognitive activities, education, and cognitive performance in aging, finding that cognitive activities in later life have a stronger association with cognitive performance across various domains, except episodic memory [16]. This suggests that engaging in cognitive activities may be more impactful than previous educational attainment in sustaining cognitive functions in older adults, although more research is needed to explore these relationships further [16]. Krause-Sorio et al. demonstrated the protective effects of three months of KY training combined with daily practice of KK on brain regions susceptible to age-related decline. The study suggests improvements in anxiety and depression among older women with subjective cognitive decline, highlighting the potential of yoga in enhancing mental and brain health in high-risk groups [11]. Soininen et al. present findings from a study on the long-term effects of multinutrient intervention in prodromal AD, showing significant improvements in cognitive function, daily activities, and brain health over three years [17]. This study emphasizes the potential for early and prolonged intervention to alter disease trajectories and improve outcomes in prodromal AD [17]. Phillips et al. explored the efficacy of a 12-week modified ketogenic diet in AD patients, noting improvements in daily functionality and quality of life alongside mostly favorable changes in cardiovascular risk factors [18]. This study suggests that ketogenic diets could be promising treatment strategies for AD, pending further research [18]. Gomez-Gallego et al. discussed the impact of active music intervention (AMI) and receptive music intervention on AD symptoms, finding that AMI significantly improves cognition, behavior, and functional state in AD patients compared to a control group. Meanwhile, receptive music intervention showed a stabilizing effect on neuropsychiatric symptoms, potentially through the evocation of positive memories and relaxation [19].

Gaitan et al. highlighted the positive association between exercise, myokine Cathepsin B (CTSB), cognition, and the modulation of lipid metabolites related to dementia. Their findings suggest that exercise-induced changes in plasma brain-derived neurotrophic factor and CTSB may enhance cognitive function in adults at risk for dementia [20]. Thota et al. reported that dietary supplementation with curcumin significantly reduced levels of IAPP and glycogen synthase kinase-3 β, suggesting a novel mechanism by which curcumin could reduce insulin resistance markers and possibly lower the risk of type 2 diabetes and AD [21]. Finally, Fonte et al. confirmed that cognitive and physical activity treatments (CT and PA) can slow the progression of cognitive symptoms in patients with MCI and AD. The study also found improvements in memory and attention, as well as cardiovascular risk factors and exercise capacity, in both MCI and AD patients, underscoring the importance of consistent treatment for these conditions [7,22,23]. Together, these studies offer a rich tapestry of evidence supporting various interventions that could potentially ameliorate the symptoms or delay the onset of AD, providing valuable insights for future research and practical applications in this field.

## Conclusions

Our systematic review provides a comprehensive analysis of multifaceted interventions aimed at delaying or preventing AD. The diverse collection of studies highlights the significant potential of lifestyle modifications, including physical exercise, dietary interventions, and cognitive activities, in enhancing cognitive function and overall well-being. Notable findings from RCT and observational studies underscore the benefits of specific diets like MIND and ketogenic diets, as well as the use of supplements such as curcumin. Additionally, holistic practices like yoga and music therapy show promising results in improving mental health and cognitive performance. Collectively, these interventions offer a holistic approach to AD prevention, emphasizing the importance of a multifaceted strategy for mitigating risk and improving quality of life for individuals at high risk or experiencing early symptoms of AD. Future research should continue to explore and refine these interventions, ensuring a robust evidence base for effective preventive measures against AD.
